# Using association rules mining to explore pattern of Chinese medicinal formulae (prescription) in treating and preventing breast cancer recurrence and metastasis

**DOI:** 10.1186/1479-5876-10-S1-S12

**Published:** 2012-09-19

**Authors:** Yanhua He, Xiao Zheng, Cindy Sit, Wings TY Loo, ZhiYu Wang, Ting Xie, Bo Jia, Qiaobo Ye, Kamchuen Tsui, Louis WC Chow, Jianping Chen

**Affiliations:** 1 School of Chinese Medicine, The University of Hong Kong, Hong Kong SAR; 2 Institute of Human Performance, The University of Hong Kong; Department of Sports Science and Physical Education, The Chinese University of Hong Kong, Hong Kong SAR; 3 Guangdong Provincial Hospital of Traditional of Chinese Medicine, Guangzhou, PRC; 4 Chengdu University of Traditional of Chinese Medicine, Chengdu, PRC; 5 The Hong Kong Society of Chinese Medicine, Hong Kong SAR; 6 UNIMED Medical Institute, Hong Kong SAR

## Abstract

**Background:**

Chinese herbal medicine is increasingly widely used as a complementary approach for control of breast cancer recurrence and metastasis. In this paper, we examined the implicit prescription patterns behind the Chinese medicinal formulae, so as to explore the Chinese medicinal compatibility patterns or rules in the treatment or control of breast cancer recurrence and metastasis.

**Methods:**

This study was based on the herbs recorded in Pharmacopoeia of the People’s Republic of China, and the literature sources from Chinese Journal Net and China Master Dissertations Full-text Database (1990 – 2010) to analyze the compatibility rule of the prescription. Each Chinese herb was listed according to the selected medicinal formulae and the added information was organized to establish a database. The frequency and the association rules of the prescription patterns were analyzed using the SPSS Clenmentine Data Mining System. An initial statistical analysis was carried out to categorize the herbs according to their medicinal types and dosage, natures, flavors, channel tropism, and functions. Based on the categorization, the frequencies of occurrence were computed.

**Results:**

The main prescriptive features from the selected formulae of the mining data are: (1) warm or cold herbs in the Five Properties category; sweet or bitter herbs in the Five Flavors category and with affinity to the liver meridian are the most frequently prescribed in the 96 medicinal formulae; (2) herbs with tonifying and replenishing, blood-activating and stasis-resolving, spleen-strengthening and dampness-resolving or heat-clearing and detoxicating functions that are frequently prescribed; (3) herbs with blood-tonifying, yin-tonifying, spleen-strengthening and dampness-resolving, heat-clearing and detoxicating, and blood-activating with stasis-resolving functions that are interrelated and prescribed in combination with qi-tonifying herbs.

**Conclusions:**

The results indicate that there is a close relationship between recurrence and metastasis of breast cancer with liver dysfunctions. These prescriptions focus on the herbs for nourishing the yin-blood, and emolliating and regulating the liver which seems to be the key element in the treatment process. Meanwhile, the use of qi-tonifying and spleen-strengthening herbs also forms the basis of prescription patterns.

## Background

Breast cancer is one of the most common malignant tumors among women, and the incidence increases every year in both developed and developing countries [1]. Every year, among the 1.2 million women diagnosed with breast cancer worldwide, 500 thousand cases die of the disease. Along with a sharp increase in life expectancy, expansion of urbanization and adaptation of western lifestyle, the increase in incidence rates is even more obvious in developing countries [2-5]. In China, the number of cases increased by 38.5% from 2000 to 2005. Compared with the early surveys in the 1990s, breast cancer accounted for the largest increase in mortality rates in 2005 [6].

Today, the standard therapies for breast cancer include surgery, chemotherapy, radiation therapy, and hormonal therapy. However, even though patients receive systemic treatment, there is still 10% to 30% chance of recurrence and metastasis. Among the patients with local recurrence, 75% to 93% will eventually develop distant metastasis with an extremely low 5-year survival rate [7,8]. Visceral metastasis is the main reason for treatment failure and cause of death. Lung, bone, liver and brain are the most common sites of distant spread of breast cancer [9,10]. Since metastasis is the main reason for cancer treatment failure, management of metastasis is the key factor for determining the prognosis of the patients [11].

Recently, the use of natural Chinese herbal medicine with anti-tumor effects is receiving more and more attention from the public [12]. In traditional Chinese medicine (TCM), the treatment and prevention of breast cancer recurrence and metastasis is a holistic approach through multi-level, multi-target and multi-channel control. CTM differs from Western medicine, which adopts ways to block a single transfer in a particular process. In comparison, Chinese medicine adopts an overall therapeutic approach to treat and prevent recurrence and metastasis, to improve the immune system of patients, and to strengthen the body’s susceptibility to diseases. Meanwhile, Chinese medicine also aims at reducing the side effects of radiotherapy and chemotherapy, reversing drug resistance and improving quality of life and survival for patients. Therefore, these unique advantages have gradually made the Chinese medicinal approach in combating breast cancer recurrence and metastasis the research focus of both the local and overseas scholars [13,14].

In Chinese medicinal therapy, experienced Chinese medical practitioners prescribe a medicinal formula—a combination of various single herbs—for the treatment of ailments. According to TCM theories, pharmacological and pharmacodynamic relationship exists among herbs, which is deemed as Chinese medicinal compatibility. The compatibility of Chinese herbal medicine has particular rules and patterns. In Chinese medicinal database, there are over ten thousand medicinal formulae which enclose complicated information. However, a well-established and orderly system for organizing the information of Chinese medicinal formulae does not exist. This implies that a large amount of implicit prescription patterns behind the formulae have not been fully disclosed [15,16].

Association rules mining is one of the methods for discovering meaningful associations or correlations between variables in large databases. It identifies frequent item sets from the data sets, and then uses these frequent item sets to form their association rules. To select meaningful rules from the set of all possible rules, minimum thresholds on support and confidence are the two important constraints. An association rule has the form *LHS*⇒*RHS*, where *LHS* and *RHS* are sets of items, and the RHS set is likely to occur whenever the LHS set occurs. One of the applications of association rules mining is to mine association rules in medical record data [17,18]. Since association rules mining is a popular and well-researched method, it can be used to investigate the Chinese herbal medicine compatibility patterns, and to reflect the interdependence and relationship between the variables. Therefore, it can provide scientific evidence for clinical applications of Chinese medicine, and thereby offer an implication for the integration of Chinese medicinal therapy with modern Western medical therapies to better treatment or prevention of breast cancer recurrence and metastasis [19]. The support supp(X) of an item set X is defined as the proportion of transactions in the data set containing the item set. It is a function used for evaluation of the potential usefulness of the rules. The confidence of a rule is defined as conf(X => Y), which can be interpreted as an estimate of the probability P (Y|X) [20].

## Methods

### Sources of literature

This study was based on Pharmacopoeia of the People’s Republic of China[21] recorded to investigate the prescription patterns of using Chinese medicine for treatment and prevention of breast cancer recurrence and metastasis. The sources of literature included the Chinese Journal Net and the China Master Dissertations Full-text Database (1990 – 2010) (Table 1). The name of each herb was used as a keyword to obtain the relevant literature, and only the literature which focused on “breast cancer”, “advanced stage of breast cancer” and/or “post-operation of breast cancer” was eligible for inclusion. According to the following inclusion and exclusion criteria, a total of 131 papers describing various medicinal formulae for clinical applications were included (96 medicinal formulae with a total of 180 Chinese herbal medicines (herbs); the total cumulative occurrences of 180 herbs appearing in 96 formulae were 1001 times). The terminologies used in this article refer to ‘WHO International Standard Terminologies on Traditional Medicine in the Western Pacific Region’, which has documented the common technical terms used in traditional medicine.

**Table 1:** Data source of the literature (1990-2010)

Database	Source	Keyword(s)	Number of literature
Chinese Journal Net Database	The herbs recorded in Chinese Materia Medica and Pharmacopoeia of the People’s Republic of China (2005 Edition) Volume I	“breast cancer” and/or “advanced stage” and/or “post-operation”"clinical research " “TCM”, "prevention and treatment of breast cancer recurrence and metastasis" and be eligible for selection criteria	121
China Master Dissertations Full-text Database	The herbs recorded in Chinese Materia Medica and Pharmacopoeia of the People’s Republic of China (2005 Edition) Volume I	“breast cancer” and/or “advanced stage” and/or “post-operation” "clinical research " “TCM”, "prevention and treatment of breast cancer recurrence and metastasis" and be eligible for selection criteria	8
China PhD Dissertations Full-text Database	The herbs recorded in Chinese Materia Medica and Pharmacopoeia of the People’s Republic of China (2005 Edition) Volume I	“breast cancer” and/or “advanced stage” and/or “post-operation” "clinical research " “TCM”, "prevention and treatment of breast cancer recurrence and metastasis" and be eligible for selection criteria	2

Among the 131 papers searched from the databases, 96 medicinal formulae were included in the study according to the inclusion and exclusion criteria

* According to the inclusion and exclusion criteria, a total of 131 papers which described various Chinese medicinal formulae for clinical applications were included 96 medicinal formulae with a total of 180 herbs. Total cumulative occurrences of 180 herbs appearing in 96 formulae is 1001 times.

### Inclusion criteria

There were five types of literature included, including literature: (1) related to clinical research on using Chinese medicine for the prevention and treatment of breast cancer recurrence and metastasis; (2) related to clinical research on using Chinese medicine for the treatment of advanced stage breast cancer; (3) related to clinical research on using Chinese medicine for the prevention of postoperative breast cancer recurrence and metastasis (especially at stage III or later when metastasis had occurred); (4) with randomized controlled trials as the study design; and (5) where the clinical study aims to prove the efficacy of experimental group with Chinese medicinal treatment over control group.

### Exclusion criteria

Literature with the following criteria were excluded: (1) small-sample-sized studies with less than 20 cases; (2) studies which primarily aimed to treat complications of operations or to reduce the side effects of chemotherapy; (3) studies without investigation into the use of Chinese medicine for the treatment and prevention of breast cancer recurrence and metastasis; (4) studies which provided only the names of formulae but without descriptions of herbal ingredients; (5) duplicate publications reporting the same group of participants; and (6) literature in which the clinical trial received a Jadad score of less than 2.

### Statistical analysis

Association rules mining is a popular and well-researched method for discovering interesting relations between variables in large databases [22]. We used the following definition for item sets and association rules. An association rule has the form LHS⇒RHS, where LHS and RHS are sets of items and the RHS set is likely to occur whenever the LHS set occurs [23].

Two parameters (support factor and confidence factors) were essential in association rules mining. With regard to support and confidence in discovering the association rules, the user shall set the minimum support (min-sup) and the minimum confidence (min-conf) as critical values providing the baselines for discovery. Only the combinations that satisfy the minimum thresholds on support and confidence were considered to mine meaningful rules. The selection of thresholds (support and confidence) was always an issue. If the minimum confidence is set too high, a lot of useful data will be missed. To find an effective drug compatibility mode, we discovered central tendency of association rules to be more obvious at the support of 0.1 and confidence of 0.6 in the two correlation analysis of these herbs (used pairs of couplet herbs) and the pairs of herbal functions. So the minimum support of 0.1 and the minimum confidence of 0.6 were specified in this study.

Based on Pharmacopoeia of the People’s Republic of China, the ingredients of Chinese medicine were listed according to the selected medicinal formulae and were organized to establish a database. The computing software Microsoft ACCESS was used as a storage tool, and then the SPSS Clenmentine Data Mining System was used as a platform to analyze the frequency and the association rules of the prescription patterns. An initial statistical analysis of the database was carried out to categorize the herbs according to their medicinal types and dosage, natures, flavors, channel tropism, and functions. The frequencies of occurrence and use were then computed based on the categorization. In additionthe associations between different functions of Chinese herbs from the formulae were also examined using the association rules mining.

## Results

### Associations between Five Properties and Five Flavors from 180 herbs prescribed in 96 formulae

The 180 herbs were categorized according to the Five Properties and Five Flavors (Table 2 & Table 3). Based on the Five properties and Five flavors theory of TCM, herbs with a warm (67 herbs, 37.22%) or cold (60 herbs, 33.33%) nature were most frequently prescribed in terms of the occurrence frequency, while herbs with a warm (appeared 90 times, 93.75%), cold (appeared 71 times, 73.96%) and neutral (appeared 82 times, 85.42%) nature were the top three prescribed herbs in terms of the frequency of use. According to the Five Flavors (Table 3), herbs that were sweet (94 herbs, appeared 93 times, 96.88%) or bitter (87 herbs, appeared 86 times, 89.58%) were the top two prescribed herbs in terms of both the frequencies of occurrence and use.

**Table 2:** Association of the herbs from 96 formulae with Five Properties (N = 180 in 96 formulae)

Five properties	Number of herbs	Occurrence frequency (%)	Number of formulae	Frequency of use (%)
Warm	67	37.22	90	93.75
Cold	60	33.33	71	73.96
Neutral	40	22.22	82	85.42
Cool	8	4.44	44	45.83
Hot	5	2.78	8	8.33

Occurrence frequency = number of herbs belonging to each category / total number of recorded herbs (i.e. 180); Frequency of use = number of formulae recording the use of the herbs / total number of selected formulae (i.e.: 96).

**Table 3:** Association of the herbs from 96 formulae with Five Flavors (N = 180 in 96 formulae)

Five flavors	Number of herbs	Occurrence frequency (%)	Number of formulae	Frequency of use (%)
Sweet	94	52.22	93	96.88
Bitter	87	48.33	86	89.58
Pungent	64	35.56	80	83.33
Salty	21	11.67	37	38.54
Sour	18	10	40	41.67

Occurrence frequency = number of herbs belonging to each category / total number of recorded herbs (i.e. 180); Frequency of use = number of formulae recording the use of the herbs / total number of selected formulae (i.e.: 96)

### Association between Channel tropism theory from 180 herbs prescribed in 96 formulae

The results of the association analysis based on channel tropism theory among 180 herbs (Table 4) showed that the top two most frequently prescribed herbs have high affinity to the liver and spleen. The herbs that have high affinity to the liver channel were used most frequently (frequency of use = 44.79%).

**Table 4:** Association of the herbs from 96 formulae with Channel tropism (N = 180 in 96 formulae)

Channel Tropism	Number of herbs	Occurrence frequency (%)	Number of formulae	Frequency of use (%)
Liver (Gall bladder)	120(13)	27.09	43(9)	44.79
Spleen (Stomach )	126(59)	28.44	42(35)	43.75
Lung (Large intestine)	77(14)	17.38	36(13)	37.50
Kidney (Urinary bladder)	64(12)	14.45	28(9)	29.17
Heart (Small intestine)	51(4)	11.51	26(4)	27.08
Triple energizers (Pericardium)	3(2)	1.13	3(2)	3.13

Occurrence frequency = number of herbs belonging to each category / total number of recorded herbs (i.e.: 180); Frequency of use = number of formulae recording the use of the herbs / total number of selected formulae (i.e.: 96).

### Frequency distribution of a single herb prescribed in Chinese medicinal formulae

There are 96 medicinal formulae with a total of 180 herbs included in this study. The total cumulative occurrence of 180 herbs appearing in 96 formulae was 1001 times. The following 13 herbs were frequently prescribed (over 20 times):

^a^ Principal function in qi-tonifying, including : Huang Qi (Radix Astragali), Bai Zhu (Rhizoma Atractylodis Macrocephalae), Gan Cao (Radix Glycyrrhizae Uralensis), Tai Zi Shen (Radix Pseudostellariae), Dang Shen (Radix Codonopsitis Pilosulae)

^b^ Principal function in spleen-fortifying and dampness-resolving, including : Fu Ling (Sclerotium Poriae Cocos), Yi Yi Ren (Semen Coicis),

^c^ Principal function in heat-clearing and detoxicating, including : Shan Ci Gu (Pseudobulbus Shancigu), Bai Hua She She Cao (Herba Hedyotidis Diffusae), 

^d^ Principal function in blood-tonifying, including: Dang Gui (Radix Angelicae Sinensis), u 

^e^ Principal function in yin-tonifying, including: Gou Qi Zi (Fructus Lycii), 

^f^ Principal function in blood-activating and stasis-resolving, including: E Zhu (Rhizoma Curcuma Phaeocaulis), and

^g^ Principal function in qi-regulating, including: Chen Pi (Pericarpium Citri Reticulatae) (Table 5).

**Table 5:** The top 13 herbs being used among the 180 herbs of the formulae in frequency

No.	Herbs (Pharmaceutical name)	Number of occurrences	Frequency of use (%)
1	Huang Qi^a^ (Radix Astragali)	60	62.50
2	Bai Zhu^a^ (Rhizoma Atractylodis Macrocephalae)	45	46.88
3	Fu Ling^b^ (Sclerotium Poriae Cocos)	39	40.63
4	Shan Ci Gu^c^ (Pseudobulbus Shancigu)	27	28.13
5	Dang Gui^d^ (Radix Angelicae Sinensis)	26	27.08
6	Yi Yi Ren^b^ (Semen Coicis)	25	26.04
7	Bai Hua She She Cao^c^ (Herba Hedyotidis Diffusae)	25	26.04
8	Gan Cao^a^ (Radix Glycyrrhizae Uralensis)	23	23.96
9	Tai Zi Shen^a^ (Radix Pseudostellariae)	22	22.92
10	Gou Qi Zi^e^ (Fructus Lycii)	22	22.92
11	Dang Shen^a^ (Radix Codonopsitis Pilosulae)	21	21.88
12	E Zhu^f^ (Rhizoma Curcuma Phaeocaulis)	20	20.83
13	Chen Pi^g^ (Pericarpium Citri Reticulatae)	20	20.83

Frequency of use = number of formulae recording the use of the herbs / total number of selected formulae

### Frequency distribution of categorized herbs according to their functions

Herbs with tonifying and replenishing (qi-tonifying, blood-tonifying, yin-tonifying and yang-tonifying), blood-activating and stasis-resolving, spleen-fortifying and dampness-resolving or heat-clearing and detoxicating functions appeared to be most frequently prescribed for the treatment and prevention of breast cancer recurrence and metastasis (Table 6). The top three functions included herbs with qi-tonifying, heat clearing and detoxicating, and blooad-activiating and stasis-resolving functions.

**Table 6:** The top 10 kinds of function herb among the 180 herbs of the formulae in frequency

No.	Functions	Number of herbs occurrences in various formulae	Occurrence frequency (%)	Number of formulae use of the herbs	Frequency of use (%)
1	Qi-tonifying	213	21.28	79	82.29
2	Heat-clearing and detoxicating	113	11.29	53	55.21
3	Blood-activating and stasis-resolving	104	10.39	56	58.33
4	Yang-tonifying	79	7.89	36	37.5
5	Spleen-fortifying and dampness-resolving	77	7.69	51	53.13
6	Yin-tonifying	70	6.99	40	41.67
7	Blood-tonifying	67	6.69	44	45.83
8	Phlegm-resolving	50	05.	37	38.54
9	Qi-regulating	48	4.8	29	30.21
10	Liver-soothing	21	2.1	19	19.79

Occurrence frequency = number of occurrences for the herbs appearing in various formulae / total cumulative occurrences for 180herbs appearing in 96 formulae ( i.e.: 1001); Frequency of use = number of formulae recording the use of the herbs / total number of selected formulae.

### Associations between pairs of herbs functions from the formulae

Association rules mining was applied to investigate the associations between pairs of herb functions from the formulae, and to examine the Chinese medicinal compatibility patterns (Table 7). The minimum support of 0.1 and the minimum confidence of 0.6 were specified.

**Table 7:** The pairs of herbal functions being used among the formulae

Herb functions (LHS, X)	Number of formulae		Herb functions (RHS, Y)	Number of formulae	Support (X) (%)	Confidence (X => Y) (%)
Blood-tonifying	44	→	Qi-tonifying	41	42.71	93.18
Yin-tonifying	40	→	Qi-tonifying	37	38.54	92.50
Phlegm-resolving	37	→	Qi-tonifying	34	35.42	91.89
Spleen-fortifying and dampness-resolving	51	→	Qi-tonifying	46	47.92	90.20
Qi-regulating	29	→	Qi-tonifying	27	28.13	93.10
Yang-tonifying	36	→	Qi-tonifying	32	33.33	88.89
Heat-clearing and detoxicating	53	→	Qi-tonifying	46	47.92	86.79
Blood-activating and stasis-resolving	56	→	Qi-tonifying	47	48.96	83.93
Blood-activating and stasis-resolving	56	→	Heat-clearing and detoxicating	35	36.46	62.50
Heat-clearing and detoxicating	53	→	Spleen-fortifying and dampness-resolving	32	33.33	60.38
Blood-tonifying	44	→	Yin-tonifying	27	28.13	61.36

The top three pairs of herbal functions with the highest confidence included the blood-tonifying paired with qi-tonifying functions (93.18%), the qi-regulating paired with qi-tonifying functions (93.10%) and the yin-tonifying paired with qi-tonifying functions (92.50%).

### Associations between pairs of couplet herbs from the formulae

Couplet herbs are two herbs used in pair to increase the therapeutic effect or reduce the toxic effect. To further examine the compatibility patterns of couplet-medicinal prescriptions, we targeted the herbs for healthy-qi reinforcement (including qi-tonifying, yin-tonifying, blood-tonifying, yang-tonifying and spleen-fortifying and dampness-resolving), and the herbs for pathogenic-factor elimination (including heat-clearing and detoxicating, blood-activating and stasis-resolving, and qi-regulating), which were frequently prescribed for the treatment and prevention of breast cancer recurrence and metastasis (Table 8). The minimum support of 0.1 and the minimum confidence of 0.6 were specified. The top three pairs of couplet herbs with the highest confidence included the Tai Zi Shen paired with Bai Zhu (86.36%), the Bai Zhu paired with Huang Qi (84.44%), and the Bai Zhu paired with Fu ling (77.78%).

**Table 8:** The commonly used pairs of couplet herbs in the formulae

Herbs (LHS, X)	Number of formulae		Herb (RHS, Y)	Number of formulae	Support (X) (%)	Confidence (X => Y) (%)
Tai Zi Shen	22	→	Bai Zhu	19	19.79	86.36
Bai Zhu	45	→	Huang Qi	38	39.58	84.44
Bai Zhu	45	→	Fu Ling	35	36.46	77.78
Bai Hua She She Cao	25	→	Fu Ling	19	19.79	76.00
Bai Hua She She Cao	25	→	Yi Yi Ren	17	17.71	68.00
Yi Yi Ren	25	→	Fu Ling	17	17.71	68.00
E Zhu	20	→	Shan Ci Gu	12	12.50	60.00

## Discussion

From the herbal perspective, breast cancer is the local manifestation of a whole-body disease, referred to as an intrinsically deficient but extrinsically excessive syndrome. Based on TCM theories, deficiency of spleen qi, inadequate source of engendering transformation, deficiency of qi and blood, and excess of phlegm-dampness are believed to be the main mechanism responsible for development of breast cancer [24,25].

### Medicinal formulae often include herbs that are sweet or bitter

The 180 herbs were classified according to the Five Flavors, and herbs that were sweet or bitter were the top two most frequently prescribed herbs in the formulae. In TCM theories, herbs that taste sweet can be used for supplementation, moderation and harmonization, referred to as tonifying and replenishing herbs. Herbs that taste bitter can be used for discharging and downbearing, referred to as heat-clearing and detoxicating herbs. However, sweet tasting herbs with spleen-strengthening functions were prescribed and used more frequently than herbs with a bitter taste for clearing heat.

### There is a close relationship between recurrence and metastasis of breast cancer and liver, and herbs for nourishing the yin-blood, emolliating and soothing the liver, and smoothing the meridians are the keys of breast cancer treatment

Breast cancer is different from the other cancer types, as the onset of this disease usually peaks at menopausal [26]. The pathological characteristic of this period is marked by exhaustion of heavenly tenth. During this period, the body suffers from yin-blood deficiency, and liver-kidney depletion. Liver is the organ for storing blood. Liver functions in free coursing, and its functions are based on sufficiency of yin-blood. In other words, the free coursing relies on the sufficiency of yin-blood stored in the liver. Therefore, not only herbs for soothing the liver and regulating qi are needed, but also the herbs for emolliating the liver blood are essential for the treatment and prevention of breast cancer recurrence and metastasis. From the association rule mining, the herbs, such as Shao Yao, Wu Wei Zi, Ji Xue Teng, Sheng Shu Di, Gou Qi Zi, Nu Zhen Zi, and Dang Gui, are used directly for blood-tonifying and liver-emolliating in treatment of breast cancer. In general, herbs for nourishing the yin-blood, emolliating the liver, soothing the liver and smoothing the meridians play a key role in breast cancer treatment.

Ample clinical research of Chinese formulae reinforces the spleen to regulate qi and soothe the liver to alleviate pain. Thus, they do not only resist tumor and strengthen the body, but also have anti-cancer effects on metastatic breast cancer [27,28].

### The use of herbs for reinforcement of healthy qi and elimination of pathogenic factors is a common Chinese medicinal combination

From the TCM perspective, the etiology of breast cancer is due to deficiency of the healthy qi, which is related to spleen qi deficiency, and liver-kidney depletion. This deficiency will result in malfunctioning of spleen, liver and kidney for transportation and transformation, and free coursing. Without the proper functioning, stagnation and obstruction of the breast collaterals will ultimately be developed and transformed into breast cancer [29].

### The use of qi-tonifying and spleen-fortifying herbs is the basis of prescription patterns for preventing breast cancer recurrence and metastasis

Restoration of healthy qi is an effective way to treat diseases and to prevent further progression. The use of qi-tonifying and spleen-fortifying herbs is to replenish the source of engendering transformation for qi and blood, and to achieve qi-tonifying, blood-replenishing and harmony of the five visceral functions. This is particularly essential for nourishing the liver and smoothing the qi movement. At the same time, spleen-strengthening and qi-replenishing herbs also have the functions for resolving dampness and dispelling phlegm. Therefore, the formulae prescribed herbs such as Huang Qi, Bai Zhu, and Fu Ling, among others.. From the association rules mining, the results showed that the combination of the herbs should also focus on the functions for qi-tonifying. The use of couplet herbs involving Huang Qi and Bai Zhu is to achieve the effects of spleen-strengthening and qi-replenishing, and dampness-drying and water-draining; the use of couplet herbs involving Bai Zhu and Tai Zi Shen is to achieve the effects of fluid-engendering and lung-moistening; the use of couplet herbs involving Bai Zhu and Fu Ling is to achieve the effects of dampness-resolving. The effectiveness of these tonifying and replenishing herbs on tumor resistance and immunity enhancement has also been proven by clinical studies [30,31].

## Conclusions

The results showed that recurrence and metastasis of breast cancer is considered to have a close relationship with liver dysfunctions. These prescriptions focus on the herbs for nourishing the yin-blood, and emolliating and regulating the liver. Strengthening of liver function seems to be the key to successful treatment. Meanwhile, the use of qi-tonifying and spleen-strengthening herbs also forms the basis of prescription patterns. It is also noteworthy that liver function is promoted by strengthening the spleen.

## Competing interests

The authors declare that they have no potential and competing interests.

## Authors' contributions

YH performed the study; JC was in charge of the study work, advice in the study design and modified in manuscript writing.

XZ, CS, ZW, TX, BJ, QY and KT equally conducted and performed the study. 

LC and WTYL gave expert advice in the study design and participated in manuscript writing.

## References

[B1] Jemal A Siegel R Ward E Hao Y Xu J Murray T Thun MJ Cancer statistics, 2008 CA Cancer J Clin 2008 58 71 96 10.3322/CA.2007.0010 18287387

[B2] Parkin DM Fernández LM Use of statistics to assess the global burden of breast cancer Breast J 2006 12 Suppl 1 S70 S80 16430400 10.1111/j.1075-122X.2006.00205.x

[B3] Hortobagyi GN de la Garza Salazar J Pritchard K Amadori D Haidinger R Hudis CA Khaled H Liu MC Martin M Namer M O'Shaughnessy JA Shen ZZ Albain KS ABREAST Investigators The global breast cancer burden: variations in epidemiology and survival Clin Breast Cancer 2005 6 391 401 10.3816/CBC.2005.n.043 16381622

[B4] Schulz M Hoffmann K Weikert C Nöthlings U Schulze MB Boeing H Identification of a dietary pattern characterized by high-fat food choices associated with increased risk of breast cancer: the European Prospective Investigation into Cancer and Nutrition (EPIC)-Potsdam Study Br J Nutr 2008 100 942 946 10.1017/S0007114508966149 18377685

[B5] Atoum MF Al-Hourani HM Lifestyle related risk factors for breast cancer in Jordanian females Saudi Med J 2004 25 1245 1248 15448776

[B6] Yang L Li L Chen Y Parkin DM Cancer Incidence and Mortality Estimates and Prediction for year 2000 and 2005 in China Chinese Journal of Health Statistics 2005 22 218 222

[B7] Vernon CC Hand JW Field SB Machin D Whaley JB van der Zee J van Putten WL van Rhoon GC van Dijk JD González González D Liu FF Goodman P Sherar M Radiotherapy with or without hyperthermia in the treatment of superficial localized breast cancer: results from five randomized controlled trials. International Collaborative Hyperthermia Group Int J Radiat Oncol Biol Phys 1996 35 731 744 10.1016/0360-3016(96)00154-X 8690639

[B8] Yuan F Xu BH The Detection and Clinical Value of Micro-metastases in Bone Marrow and for Patients with Breast Cancer Foreign Medical sciences (cancer section) 2002 29 283 286

[B9] Gonzalez-Angulo AM Morales-Vasquez F Hortobagyi GN Overview of resistance to systemic therapy in patients with breast cancer Adv Exp Med Biol 2007 608 1 22 10.1007/978-0-387-74039-3_1 17993229

[B10] Mundy GR Metastasis to bone: causes, consequences and therapeutic opportunities Nat Rev Cancer 2002 2 584 593 10.1038/nrc867 12154351

[B11] Héry M Delozier T Ramaioli A Julien JP de Lafontan B Petit T de Ghislain C Weber B Bonneterre J Teissier E Natural history of node-negative breast cancer: are conventional prognostic factors predictors of time to relapse? Breast 2002 11 442 448 10.1054/brst.2002.0462 14965709

[B12] Cohen I Tagliaferri M Tripathy D Traditional Chinese medicine in the treatment of breast cancer Semin Oncol 2002 29 563 574 10.1053/sonc.2002.50005 12516039

[B13] Mukherjee AK Basu S Sarkar N Ghosh AC Advances in cancer therapy with plant based natural products Curr Med Chem 2001 8 1467 1486 11562277 10.2174/0929867013372094

[B14] Hsiao WL Liu L The role of traditional Chinese herbal medicines in cancer therapy--from TCM theory to mechanistic insights Planta Med 2010 76 1118 1131 10.1055/s-0030-1250186 20635308

[B15] Lu W Wang YF Wu ZH System for mining association rules from traditional-chinese medicine database Journal of Zhejiang University (Engineering Science) 2001 35 22 25

[B16] Chen W Feng LY Yan SQ Study on Innovation of Method and Theory of Prescription Compatibility J Anhui Traditional Chinese Medical College 2008 27 9 11

[B17] Doddi S Marathe A Ravi SS Torney DC Discovery of association rules in medical data Med Inform Internet Med 2001 26 25 33 11583406

[B18] Stilou S Bamidis PD Maglaveras N Pappas C Mining association rules from clinical databases: an intelligent diagnostic process in healthcare Stud Health Technol Inform 2001 84 1399 1403 11604957

[B19] Zhou X Chen S Liu B Zhang R Wang Y Li P Guo Y Zhang H Gao Z Yan X Development of traditional Chinese medicine clinical data warehouse for medical knowledge discovery and decision support Artif Intell Med 2010 48 139 152 10.1016/j.artmed.2009.07.012 20122820

[B20] Laxminarayan P Alvarez SA Ruiz C Moonis M Mining statistically significant associations for exploratory analysis of human sleep data IEEE Trans Inf Technol Biomed 2006 10 440 450 10.1109/TITB.2006.872065 16871710

[B21] Pharmacopoeia of the People’s Republic of China 2005 I Chemical Industry Press PRC

[B22] Agrawal R Imieliński T Swami A Mining association rules between sets of items in large databases Proceedings of the 1993 ACM SIGMOD international conference on Management of data: 26-28 May 1993 Washington, D.C

[B23] Creighton C Hanash S Mining gene expression databases for association rules Bioinformatics 2003 19 79 86 10.1093/bioinformatics/19.1.79 12499296

[B24] Chen JP Chiu SW Chok HP Wang Z Zheng X Lin Y Prevention strategies for recurrence and metastasis of breast cancer by Chinese medicine Global Traditional Chinese Medicine 2011 4 125 128

[B25] Xiong HS Xu JZ Experience of professor Zhen weiqin of treating breast cancer Journal of Emergency in Traditional Chinese Medicine 2010 19 805 806

[B26] Lin Y Liu XY Chen QJ Risk factors assessment and prevention strategies for breast cancer with traditional Chinese medicine Traditional Chinese Medicine of Liaoning University 2008 10 6 9

[B27] Wang ZY Li AY Clinical observation of liver metastasis of mammary cancer treated with CAF chemotherapy plus traditional Chinese drugs Clinical Journal of Medical officer 2003 31 22 24

[B28] Sun H Li PP Xie YT Impact of Shu Gan Liang Xue decoction combined with tamoxifen on the growth and estrogen receptor expression in human breast cancer cell line MCF-7 J China Cancer 2005 14 607 610

[B29] Chen Q Si T Guan R Xu R Xu B Dai Y Liu P Lin Y Clinical study of 545 cases of mammary cancer patients in postoperative consolidation for syndrome and classification analysis Beijing Journal of Traditional Chinese Medicine 2008 27 590 592

[B30] Guo Y Yao QH Yang WH Luo T Clinical research on the method of replenishing qi to invigorate spleen interfering in the spleen deficiency syndrome that caused by chemotherapy to mastocarcinoma patients Chinese archives of Traditional Chinese Medicine 2007 25 2443 2446

[B31] Huang ZQ Tian HQ Lang JM Liang GW Chen WJ Jian KP Effect of astragalus Injection on life quality, tumor necrosis and immunity in the patients with advanced carcinoma Henan University of Chinese Medicine 2004 112 28 29

